# Modeling enzootic raccoon rabies from land use patterns - Georgia (USA) 2006-2010

**DOI:** 10.12688/f1000research.2-285.v2

**Published:** 2014-03-18

**Authors:** John E. Duke, Jesse D. Blanton, Melissa Ivey, Charles Rupprecht

**Affiliations:** 1 Institute of Public Health, Georgia State University, Atlanta, Georgia 30303, USA; 2 Poxvirus and Rabies Branch, United States Center for Disease Control and Prevention, Atlanta, Georgia 30329, USA; 3 Georgia Department of Public Health, Atlanta, Georgia 30303, USA

## Abstract

We analyzed how land-use patterns and changes in urbanization influence reported rabid raccoons in Georgia from 2006 - 2010.  Using Geographical Information Systems and rabies surveillance data, multivariate analysis was conducted on 15 land-use variables that included natural topography, agricultural development, and urbanization to model positive raccoon rabies cases while controlling for potential raccoon submission bias associated with higher human population densities.  Low intensity residential development was positively associated with reported rabid raccoons while a negative association was found with evergreen forest.  Evergreen forests may offer a barrier effect where resources are low and raccoon populations are not supported.  Areas with pure stands of upland evergreen forest might be utilized in baiting strategies for oral rabies vaccination programs where fewer or no baits may be needed.  Their use as a barrier should be considered carefully in a cost-effective strategy for oral rabies vaccination (ORV) programs to contain the western spread of this important zoonotic disease.

## Introduction

**Figure 1.**
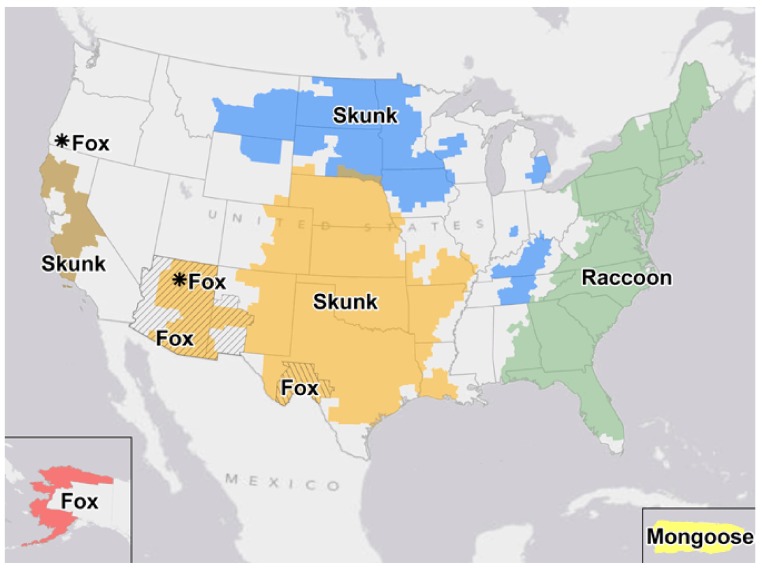
Reservoir species for rabies across the USA.

Here we use Geographical Information Systems (GIS) analysis to exhibit how both natural topography and urbanization potentially affect the dynamics of a zoonotic disease in its reservoir species. Today, the primary reservoir for rabies in Georgia, and, in fact, the entire eastern part of the USA, is the raccoon ( *Procyon lotor*). Although dogs previously served as the main reservoir for rabies throughout the USA, dog rabies virus *variants* in the USA have been eliminated through persistent vaccination programs and policies ^1^. Rabies virus has evolved to adapt to a wildlife meso-carnivore in a population that can be defined by geographical regions where transmission is sustained ( Figure 1) ^2^. Over the second half of the last century, the eastern raccoon rabies variant has spread northward and, in Georgia, the raccoon rabies enzootic has been established for over 50 years ^3^.

Infection from the reservoir species to another mammalian host can, and still does occur, but the *non*-reservoir animal most likely becomes a dead-end host ^4^. Cases of rabies in Georgia in non-reservoir wildlife, such as foxes, coyotes and skunks, and in domesticated animals, such as dogs, cats, and farm animals, can almost always be traced either to the raccoon variant, or to one of the circulating variants among several bat species, through DNA molecular typing ^5^. Using Georgia Department of Public Health laboratory data on raccoon specimens submitted for rabies testing, and the US Geological Survey Land Cover Database, we report a model to predict rabid raccoon cases from land use patterns.

### Raccoon rabies epidemiology

It is well documented that higher densities of raccoon populations exist in urbanized areas than in more natural habitats, and, that the highest densities are found at the urban-rural interface ^6– 9^. An opportunistic omnivore, eating anything from crayfish in creeks to acorns, bird eggs, garden vegetables and even garbage, raccoons have been essentially subsidized by urbanization by the creation of an edge ecosystem. An edge ecosystem is created when habitat is broken up. This can occur naturally, such as disturbance by fire, or can be part of the geomorphology, such as the demarcation between uplands and wetlands. Or it can occur when development creates a new transition between forest and open space. Although some of the open space is replaced with impervious surfaces, i.e. roads and rooftops, higher raccoon populations can be sustained because they have increased access to food resources, and fewer predators, while still using the adjacent natural habitat for protective cover ^10^.

**Figure 2.**
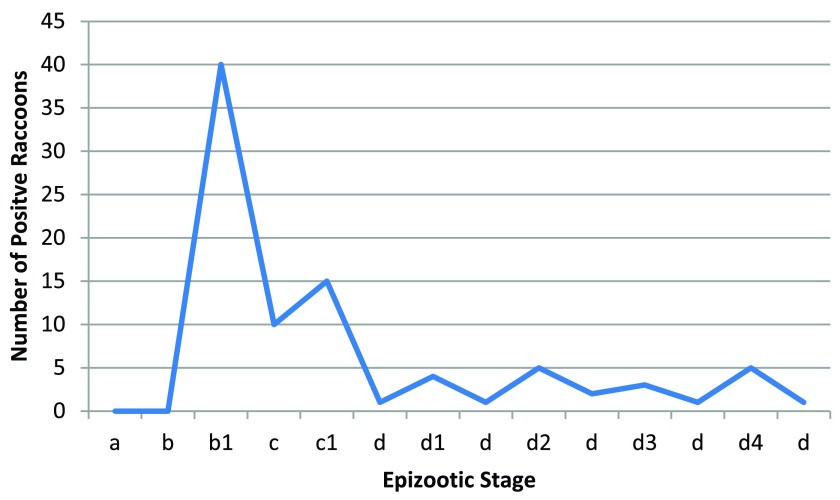
Schematic of the temporal stages of a typical county in New York during the raccoon rabies epizootic from 1992 to 2000. Stage a: Pre-raccoon variant – rare cases in raccoons; cases of raccoon rabies might spill over *from* other wildlife. b1: initial epizootic of raccoon variant rabies as it moves through the county. c1: sequential epizootic. d1: sequential inter-epizootics. (Adapted from Gordon *et al.*, 2004 ^13^).

This raccoon ecological theory, and the way it relates to land use patterns, has been supported in numerous studies where raccoon rabies epidemics occur during the initial introduction of rabies into naïve raccoon populations. These studies have described “sensational” epizootics so fulminating that natural barriers such as rivers can only slow them down ^11, 12^. Once the infection becomes enzootic in a raccoon population, large outbreaks rarely occur and the disease becomes incessant ( Figure 2) ^13^.

### Mitigating the passive public health database

The number of raccoons submitted for rabies testing listed by county and month was acquired from the Georgia Department of Public Health for a five-year period from 2006 to 2010. This database only represents results from raccoons involved in human or domestic animal exposure incidents that are submitted to the Georgia Public Health Laboratories (GPHL). Testing is done solely to inform medical professionals how to proceed with rabies prophylaxis for potential human exposure cases. The presence of rabies virus in the raccoon specimens is tested by direct fluorescent-antibody staining of brain tissue. [ http://www.cdc.gov/rabies/diagnosis/direct_fluorescent_antibody.html].

**Figure 3.**
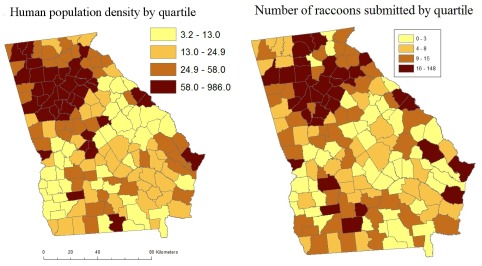
The population densities of 159 Georgia counties by quartile from the 2010 census as compared to the number of raccoons submitted per county for rabies testing by quartile from 2006–2010.

The mosaic of urban and suburban development poses challenges to this type of surveillance of the raccoon rabies reservoir because higher human population densities bias reporting to passive public health surveillance systems ( Figure 3); detection of positive cases in developed areas is likely increased due to the probability of more human-raccoon and domestic animal-raccoon interactions ^14^. Due to potential confidentiality issues concerning the person exposed, this data is aggregated and reported at the county level. The number of raccoons submitted for testing closely parallels the human population densities.

**Figure 4.**
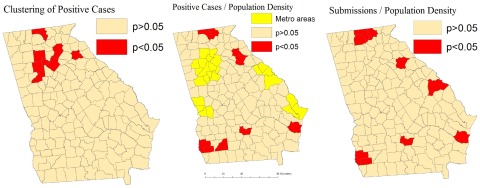
Significant clustering of positive cases occurs mostly in the metro Atlanta area where there are also high submission rates. The clustering of positive cases is mostly away from major population centers when analyzed as a rate of population density. Clustering of submissions/density closely parallels positive cases/density.

Prior to 2006, only positive cases were reported by GPHL. Because the 2006–2010 GPHL dataset reported both positive and negative submissions, there was opportunity to possibly mitigate the surveillance bias to some extent by incorporating a standardizing variable. The Getis-Ord Gi statistical tool in GIS (ESRI ArcMap 10) was used to exhibit spatial clustering of counties with higher or lower than expected number of positive cases, positive cases per person per square kilometer, and the number of submissions per person per square kilometer ( Figure 4) ^15^.

When the number of positive cases is put at a rate of population density, the clustering disperses away from the metropolitan areas. However, because our model counted the number of positive cases as the outcome variable, we wanted to use some form of the *submissions* data as the “weighted” standardizing variable on the right side of the regression model to normalize counties.

Table 1 summarizes the human demographic and GPHL testing data. There were forty-six counties that were inclusive in the upper quartile for positive cases. Forty counties were inclusive in the upper quartile for the number of submissions for testing, as well as all other independent land use variables. The high odds ratio for both submissions, and population density, confirms that as there is more testing, there are more positive cases; it is a matter of more intense surveillance, and does not necessarily reflect the pattern of the rabies reservoir in the raccoon. However, when density is used as the denominator to analyze the effect of rate of submissions on disease predictability, the odds ratio falls (3.1). The drop in the odds ratio, though still significant, could reflect two possibilities: that there truly are higher raccoon densities in more populated areas and/or that the database bias has been mitigated to some extent.

**Table 1.** Human demographic data, number of submissions for testing, and positive raccoon cases from 2006–2010 for 159 Georgia counties. Sub/density is the weighted variable used in the regression model. High enzootic is the number of counties in the upper quartile of positive cases (≥ 8).

				Enzootic	Enzootic			
Variable	Median	Upper quartile	Range	high	low	OR	95% CI	p value
**density** ^a^	24.9	58	3.2–986.1	23	17	**5.6**	**2.6–12.3**	**<0.001**
Submissions	8	15	0–148	35	5	**68.7**	**22–211**	**<0.001**
Positive	4	8	0–50	**---**	**---**	**---**	**---**	**---**
**Sub/density** ^b^	0.238	0.432	0–1.66	19	21	**3.1**	**1.5–6.6**	**0.003**

^a^people per km ^2^

^b^number of submitted raccoons for testing per person per km ^2^

OR: odds ration

CI: confidence interval

### Land use/land cover and human demographic analysis

Using GIS, we extracted fifteen land use/land cover categories from the National Land Cover Database 2006 [ http://www.mrlc.gov/nlcd06_leg.php]. Variable construction was based on the method of Jones *et al.* 2003 ^16^, to define low intensity residential, high intensity residential and commercial land use. These three development variables are determined from measuring the impervious surface, and constitute less than 50% (combining “open space” and “low intensity”), 50 – 79% (“medium intensity”), and 80% or greater impervious surface area (“high intensity”), respectively. The agricultural variable was also constructed on the method of Jones *et al.* 2003, and is a combination of pasture/hay and cultivated crops. Both, demographic data, and land area of each county, were obtained from the 2010 US Census [ http://quickfacts.census.gov/qfd/states/13000.html].

There were 1011 rabid raccoons from a total of 2064 raccoon specimens submitted from 150 counties over the five year period. A univariate analysis was conducted for all variables, including land-use data, human population density, and the number of rabid raccoons (outcome variable), by dichotomizing at the upper quartile to determine crude odds ratios (OR).

Urbanization variables (“Low intensity res”, “High intensity res”, and “Com/Ind/Trans”) had the highest crude odds ratios among all the land use variables examined ( Table 2). Shrub and the woody wetlands had similar protective odds ratios (0.2–0.3; CI 0.1–0.9) while evergreen forest had the lowest crude odds ratio at 0.04 (CI 0.006–0.3).

**Table 2.** Crude odds ratios for land-use patterns associated with relative risk of high enzootic raccoon rabies (≥8 positive cases) within 159 Georgia counties.

				Enzootic	Enzootic			
Variable	Median ^a^	Upper quartile ^a^	Range ^a^	high	low	OR	95% CI	p value
Open water	0.79	1.66	0.4–29.6	14	26	1.5	0.7–3.1	0.329
**Low intensity res**	58.1	98	2.8–44.5	26	14	**9.2**	**4.1–20.6**	**<0.001**
**High intensity res**	0.28	0.70	0.1–8.0	25	15	**7.8**	**3.5–17.2**	**<0.001**
**Com/Ind/Trans**	0.7	2.3	.03–4.8	23	17	**5.6**	**2.6–12.3**	**<0.001**
**Barren**	0.22	0.57	.08–1.1	17	23	**2.3**	**1.1–4.9**	**0.029**
**Shrub**	2.84	4.72	0.8–9.1	5	35	**0.3**	**0.1–0.7**	**0.008**
Deciduous forest	21.35	30.17	4.1–46.1	13	27	1.3	0.6–2.7	0.566
**Evergreen forest**	21.54	31.05	11.5–12.6	1	39	**0.04**	**.006–0.3**	**<0.001**
Mixed forest	3.02	5.08	1.1–9.6	10	30	0.8	0.3–1.7	0.528
Agricultural	16.72	25.32	9.0–29.7	16	24	2.0	0.9–4.2	0.075
Grasslands	6.15	8.59	3.9–4.1	11	29	0.9	0.4–2.0	0.818
**Woody wetlands**	6.53	16.03	2.9–29.3	4	36	**0.2**	**0.07–0.6**	**0.002**
**Herbaceous wetlands**	0.27	1.21	.01–23.7	6	34	**0.3**	**0.1–0.9**	**0.025**

^a^percentage of land use

OR: odds ratio

CI: confidence interval

The independent variables with a p-value less than 0.1 were selected to build a regression model for predicting the number of rabid raccoons. Variables were eliminated through a manual backwards stepwise regression. Although the selection of independent variables (percentage land-use) to begin the model was determined with dichotomized data (odds ratios), the regression model predicted the number of rabid cases from continuous data. Potential autocorrelation between variables in the model were considered in the backwards stepwise regression by first removing co-variables with a correlation coefficient great than 0.6; the co-variable with the highest p-value was eliminated. Because of high variance among the counties, including zeros, in the number of rabid raccoons, both Poisson and negative binomial distributions were evaluated for the regression analysis ^17^.

A negative binomial distribution was chosen for the final regression model after comparing the goodness of fit statistics (AIC=795, deviance/degrees of freedom=1.1, Pearson chi-square/degrees of freedom=0.96) to a model using the Poisson distribution (961, 3.2, and 3.2, respectively). The final negative binomial regression model included:

**+ 1.9(Sub/density)**[p<0.001] **– 1.8(Evergreen forest)**[p=0.02] **– 52.2(Com/Ind/Trans)**[p<0.001] **+ 11.5(Low intensity res)**[p<0.001] **+ 43.5(Barren)**[p=0.019] as predictors of total positive raccoon rabies cases.

Modeling Enzootic Raccoon Rabies from Land Use Patterns - Georgia (USA) 2006-2010 Data Set LIR=Low intensity residential, HIR=High intensity residential, Com=Commercial, Dec For=Deciduous Forest, Evg=Evergreen, Grass=Grasslands, Ag=Agricultural, Ed Wet-Woody Wetlands, Herb=Herbaceous, Sub=Submissions, Pos=Positive. Land use columns are given in square kilometers and percentages

## Conclusion

This study supports the hypothesis that the majority of rabid raccoons are associated with low intensity urbanized areas. If raccoon populations were not “subsidized” and the raccoon densities were allowed to remain at normal carrying capacities, rabies as a reservoir in raccoons might possibly eradicate itself. Our finding that upland evergreen forests are inversely associated with the presence of rabid raccoons supports this possibility too, and, coincides with epidemiological theory that a population threshold is needed to maintain transmission of any infectious disease ^18^. For example, pine forests that are managed for timber production generally have lower raccoon utilization as compared to other forested habitats ^19^. Therefore, compelling evidence exists that the pure stands of managed pine forests in western and southern Alabama have been attributed as being the major barrier to the spread of the raccoon enzootic further west into Mississippi ^20^.

The finding that commercial development was a positive predictor of raccoon rabies in the univariate analysis but had a negative coefficient in the final model suggests that, alone, commercial development is a co-associate of the low intensity urbanization phenomenon. Since the commercial development variable remained in the regression model with a negative coefficient however, it seems that as mixed land patterns give way to highly impacted landscapes, with high levels of impervious surface, there reaches a point at which protective cover falls below a minimal threshold.

The finding that barren land-cover is a predictor of positive raccoon rabies cases has no precedence in the literature, and contributed very little to the model, even with a high coefficient, due to the low proportion of this land type within our entire study area (0.34%). Barren land can mean rock that is exposed either naturally, or from strip mines and gravel pits. There seems to be no ecological reason for these land types to be associated with raccoons. However, “barren” also consists of land with less than 15% vegetative cover that has been temporarily cleared in preparation for development (future urbanization) and can include landfills (food resources) [ http://www.hq.nasa.gov/iwgsdi/Barren_Land.html].

The submissions/ *population density* variable was an attempt to normalize all counties in the multivariate model. We added this variable into the regression equation because the crude odds ratio for submissions/ *population* (alone) was higher (4.1). The lower odds ratio using density as the denominator indicates that testing bias is better controlled. The odds of a county being in the upper quartile of percent evergreen forest coverage and having submitted *fewer* raccoon specimens for testing per person per square kilometer (the standardized variable) as compared to any other county is 1.1 (CI: 0.5–2.6 CI). This insignificant odds ratio means that although there are fewer submissions for testing in those counties with high coverage of evergreen forest as compared to other counties there is no difference in submission *rate* among the counties, and surveillance bias has been mitigated.

Finally, our study model has implications for the enzootic areas along an oral rabies vaccination (ORV) zone. This vaccination zone lies along the entire western edge of the eastern raccoon rabies reservoir (see Figure 1). Oral vaccination baits are dropped along roadways and from planes along this front to prevent the westward spread of the raccoon rabies variant. Evidence here suggests support for possibly using few or no baits in a pure stand of upland evergreen forest, at least in the southern US. In a similar fashion to dropping baits along a river in a riparian area, baits could be dropped up to the edge of an upland evergreen forest, as those raccoons in a pure upland evergreen forest may primarily use adjacent habitats for their resource needs ^21^. The baits saved through this procedure could be distributed elsewhere with no loss in control effort. Conversely, bait density could be increased in areas that have been fragmented due to low intensity development.
