# Supplementary material for: Modeling enzootic raccoon rabies from land use patterns - Georgia (USA) 2006-2010
Source: F1000Res. 2014 Mar 18;2:285. Originally published 2013 Dec 27. [Version 2] doi: 10.12688/f1000research.2-285.v2 (PMC3962005; doi:10.12688/f1000research.2-285.v2)
Supplement: Peer review history

# Peer review: Referee response for version 1

**Published:** 2014-03-06

**Authors:** Nicole Gottdenker

**Affiliations:** 1 Department of Pathology, University of Georgia, Athens, GA, USA

**General comments:**

This article provides an interesting analysis of the available data on raccoon rabies from a public health database in order to evaluate how land use impacts raccoon rabies transmission. In particular, results show that evergreen forests (likely managed pine stands) are negatively associated with raccoon rabies virus- a finding that has potential implications for habitat types used in the delivery of oral rabies vaccine. 

- Can you justify/explain why you did backwards stepwise regression to eliminate variables versus other approaches for data/model analysis of raccoon rabies predictors?  
   
- Can you find some more information from the literature for your discussion that evaluates the relative raccoon population densities/abundances in the types of habitats that you study, even if from other states, in addition to reference 19?
   
- Also, can you discuss potential differences in spatial aggregation in raccoon populations relative to the habitat types evaluated in your study?

**Minor comments:**

- When describing table 1 in the fourth paragraph under “Mitigating the passive public health database”, change ‘odds ration’ to ‘odds ratio’.
   
- I suggest eliminating the first sentence in your conclusion.

I have read this submission. I believe that I have an appropriate level of expertise to confirm that it is of an acceptable scientific standard.

# Response

**Published:** 2014-03-10

**Authors:** John Duke

**Affiliations:** Corvax Company, LLC, USA

Thank you so much for your comments.

While the different statistical methodologies were discussed among the authors and others, the final decision was that, in our opinion, backwards stepwise regression was the most comprehensive way to include any independent variable that might influence reported cases.

The National Land Cover Database provides land classification at such a high resolution that it offers more land classes than the literature currently uses to describe raccoon natural history.  Current literature describes raccoon abundance/density in a little more broader/general terms.  It seems that the Landsat technology used to classify land patterns can overpower the ability to provide on-the-ground, population data in wildlife.  There are probably multiple reasons for this gap in information, including prioritization and practicality of research goals.  

The minor comments were accepted and the article revised.

# Peer review: Referee response for version 1

**Published:** 2014-03-03

**Authors:** Colleen Jonsson

**Affiliations:** 1 Department of Microbiology and Immunology, University of Louisville, Louisville, KY, USA

I find the content and data of this article acceptable. I would add a few more sentences about the statistical approach to provide more background to those not familiar with a crude odds ratio

I have read this submission. I believe that I have an appropriate level of expertise to confirm that it is of an acceptable scientific standard.

# Response

**Published:** 2014-03-10

**Authors:** John Duke

**Affiliations:** Corvax Company, LLC, USA

Thank you so much for your comments.

We felt that a reader could quickly look up the term "crude odds ratio" to have enough understanding to proceed.  I hope we made it clear that the crude odds ratio is a dichotomized, sort of "quick and dirty" way to decide which variables to use before proceeding with continuous data in the regression model.
